# Hijacking of the Host’s Immune Surveillance Radars by *Burkholderia pseudomallei*

**DOI:** 10.3389/fimmu.2021.718719

**Published:** 2021-08-11

**Authors:** Vanitha Mariappan, Kumutha Malar Vellasamy, Muttiah Barathan, A. S. Smiline Girija, Esaki M. Shankar, Jamuna Vadivelu

**Affiliations:** ^1^Center for Toxicology and Health Risk Studies, Faculty of Health Sciences, Universiti Kebangsaan Malaysia, Kuala Lumpur, Malaysia; ^2^Department of Medical Microbiology, Faculty of Medicine, Universiti Malaya, Kuala Lumpur, Malaysia; ^3^Department of Microbiology, Saveetha Dental College and Hospitals, Saveetha Institute of Medical and Technical Sciences (SIMATS), Chennai, India; ^4^Infection Biology, Department of Life Sciences, Central University of Tamil Nadu, Thiruvarur, India

**Keywords:** *Burkholderia pseudomallei*, immunology, melioidosis, pathogenesis, virulence

## Abstract

*Burkholderia pseudomallei* (*B. pseudomallei*) causes melioidosis, a potentially fatal disease for which no licensed vaccine is available thus far. The host-pathogen interactions in *B. pseudomallei* infection largely remain the tip of the iceberg. The pathological manifestations are protean ranging from acute to chronic involving one or more visceral organs leading to septic shock, especially in individuals with underlying conditions similar to COVID-19. Pathogenesis is attributed to the intracellular ability of the bacterium to ‘step into’ the host cell’s cytoplasm from the endocytotic vacuole, where it appears to polymerize actin filaments to spread across cells in the closer vicinity. *B. pseudomallei* effectively evades the host’s surveillance armory to remain latent for prolonged duration also causing relapses despite antimicrobial therapy. Therefore, eradication of intracellular *B. pseudomallei* is highly dependent on robust cellular immune responses. However, it remains ambiguous why certain individuals in endemic areas experience asymptomatic seroconversion, whereas others succumb to sepsis-associated sequelae. Here, we propose key insights on how the host’s surveillance radars get commandeered by *B. pseudomallei*.

## Introduction

Melioidosis is a systemic infectious disease caused by *Burkholderia pseudomallei* (*B. pseudomallei*), a Gram-negative environmental saprophyte commonly found in wet soils and contaminated waters of endemic areas (1). Melioidosis is sporadic in several countries but endemic in Southeast Asia, India and northern Australia, and some parts of the tropical and sub-tropical world (2, 3). The major route of entry is *via* inhalation or inoculation through skin breaks exposed to contaminated soil or water (4). However, laboratory-acquired cases, person-to-person spread (5), sexual transmission (6), breast milk (7) and mother-to-child transmission (8) have also been reported. The incubation period varies from less than a day to 21 days and can extend to several months or years (9, 10).

Melioidosis can occur either as primary syndrome or as a component of sepsis. The infectious melioidosis disease manifestation appears as abscess formation in visceral organs such as lungs, liver, spleen and soft tissue (11). Occasionally, infections can remain sub-clinical, while others can develop acute or chronic disease or can even progress to fatal sepsis (12). Symptoms extending for ≥2 months is defined as chronic melioidosis that usually occurs in ~10% of infected individuals (10, 13). Melioidosis relapse is relatively common due to failure by the host to eradicate *B. pseudomallei* during the primary stages of infection, especially in the immunocompromised, and even after prolonged antimicrobial therapy. The overall relapse rate can range between 15% and 30% in severe melioidosis (14, 15). Dormant sub-clinical infections are also recognized in several cases, whereby it could stimulate to cause disease, typically in association with an inter-current illness, classically with lung disease, surgical procedure or trauma. Additionally, late-onset diabetes, renal failure and immunosuppressive therapy could also contribute to reactivation (16, 17). The aspects that affect the disease exposition and presentation are still unknown. Nevertheless, alterations in the virulence of infecting strains together with host immune-competence could likely contribute to disease prognosis (18).

## Virulence Arsenal

*B. pseudomallei* is an extra-ordinarily adapted facultative intracellular parasite that can adhere, invade, survive and replicate within pulmonary (airway) epithelial and phagocytic cells. For most pathogenic bacteria, the first and foremost critical step involves the establishment of infection predominantly *via* cell adhesion. For *B. pseudomallei*, cell adhesion is mediated by specific membrane proteins such as extracellular adherence protein, flagellin and adhesin that are regulated by temperature (19). On the other hand, type IV pili, flagella and motility also play a role in adhesion (20). Following adhesion *B. pseudomallei* tend to invade the epithelial cells *via* specific virulence systems viz., type III (T3SS) and VI (T6SS) secretion systems (21). Miscellaneous virulence factors that appear to play a role in virulence include a type II secretion system (T2SS) (22), quorum sensing (23), flagellin (24), type IV pili (20), lipopolysaccharide (LPS) and capsule (25), phospholipase C (26), lactonase family protein A (27). Following invasion, *B. pseudomallei* intracellular survival in the host cell explains the ability for latency. The bacterium can evade from digestion within endocytic vacuoles by entering into the cytoplasm, causing membrane protrusions by induction of actin polymerization at one of the bacterial poles (28). On the other hand, the bacterium also displays colony morphology variations leading to formation of small colony (SCVs) or mucoid variants, that can also produce biofilm attributing to high level resistance to antimicrobial agents (29). Besides, *B. pseudomallei* can survive hostile environmental conditions including differences in pH, lack of nutrients, low or high temperatures and disinfectant detergents and antiseptic solutions. Likewise, *B. pseudomallei* can outgrow several lines of cellular as well as humoral defenses. Further, *B. pseudomallei* produces several enzymes and toxins, including hemolysins, proteases, lipases, lechithinase, cytotoxic exolipids, catalase, perioxidase, and superoxide dismutase (SOD) (3). It has become evident that *B. pseudomallei* can resist several anti-microbial peptides, complement proteins, lysosomal defensins and cationic peptidases providing itself an edge to survive adverse conditions (30).

## Host-Pathogen Interactions

Infection of the host tissue by a pathogenic bacterium involves a complex interplay between a wide array of factors that determine disease prognosis. A pathogen must acquire necessary nutrients from the host, and must almost always produce virulence factors to inflict cell damage, also evading the host’s immune armamentaria in parallel. Understanding the communication of a bacterium with the host environment and the host’s response during infection has been explored at the whole-genome level. High-density host DNA microarray analysis in response to pathogenic infection offers a resourceful tactic to closely study the pathogen-host interactions. In the past few years, numerous studies have been carried out using DNA microarray analysis to identify and determine the regulation of host responses to bacterial infection (31, 32). The aptitude to review the responses of a great subsection of the host genome and to explore the patterns and trends between the profiles across diverse subsets of hosts and pathogens, permits numerous essential and ultimate questions to be addressed in regard to the foundation of pathogen recognition, the structures of host-pathogen interaction, and the host defense and microbial virulence mechanisms (33, 34).

Largely, host cells exhibit a transcriptional program that produces an intracellular anti-infective state, in response to bacterial infection, specifically relating type I interferons, rendering neighboring cells refractory to infection. Using a cDNA microarray, a powerful approach to understand host-pathogen interactions, the gene expression profiling is monitored. Studies suggest that genome-wide expression analyses, especially at the molecular level, required to comprehend the pathology instigated by *B. pseudomallei* (35). Host gene expression and regulation profiling of cells infected with *B. pseudomallei* would likely unveil the mechanisms adopted by the host in reply to bacterial infection and provide key clues to evasion of host defenses thereby necessitating pathogenic survival and growth within cells. This would also aid to identify newer therapeutic targets and strategies (35).

Chin et al. (2010) (36) established an experimental mice model for acute phase melioidosis and employed microarray approach to explore the global host-pathogen interactions. The study revealed that genes associated with immune responses, stress responses, cell cycle regulation, proteasomal degradation, cellular metabolism and signal transduction pathways were markedly altered. Of note, genes associated with ‘core host immune responses’ and acute inflammatory responses were upregulated signifying the onset of host tissue injury.

Vellasamy and colleagues (2016) (37) explored the transcriptional responses of human lung epithelial cells upon exposure to early infection of live *B. pseudomallei* and its secreted proteins using a microarray platform. The investigation showed that the host carbohydrate metabolism and apoptosis were activated, and simultaneously, amino acid metabolism and innate immune responses were curbed in the host cells exposed to both live bacteria as well as its secreted proteins. The authors concluded that early exposure prior to establishment of infection could be associated with primary activation of host genes engaged with bacterial spread from lungs to other target organs or to evade from likely sensing by macrophages.

Similarly, Rao et al. (2020) (38) carried out transcriptomic profiling of *B. pseudomallei*-infected human lung epithelial cells with an aim to describe the cellular responses during early stages of infection. Their findings showed that the PERK-mediated unfolded protein response (UPR) was enhanced in the signaling pathway of protein processing in the endoplasmic reticulum. Other genes related to inflammatory responses, cell migration, and apoptosis were also altered. Overall, findings from both Rao et al. (2020) (37) and Vellasamy et al. (2016) (38) provided significant primary insights into the close interaction between *B. pseudomallei* and lung epithelial cells, which can be further explored to elucidate the cellular mechanisms of *B. pseudomallei* infection.

However, recently, Heacock-Kang et al. (2021) (39) established *B. pseudomallei* mono-cells transcriptomic profiling *via* host cell infection at distinct stages (vacuole entry; cytoplasmic escape and replication; and membrane protrusion, promoting cell-to-cell spread) in order to yield pathophysiological insights through a ‘TRANSITomic’ approach. The *B. pseudomallei* ‘TRANSITome’ divulged dynamic gene expression alteration *via* the various stages of host cell infection. Several genes that are essential for pathogenesis and virulence were identified, including the secretion systems. Overall, the *B. pseudomallei* ‘TRANSITome’ offered mono-cell transcriptomics data permitting high-resolution understanding of host-pathogen interactions.

## Front-line Barricades Against *B. pseudomallei*

Innate immunity is described to fight almost against any infection and deploys immune cells at the front-line in respond to microbial pathogens that endeavor to breach into the host boundaries (40). As a general description, the innate immune system comprises all the host defense aspects including physical, biochemical and microbial components. The physical barriers include epithelial cell layers in addition to the respiratory, gastrointestinal, and the genitourinary tracts. Chemical and biological barriers comprise of complement proteins and antimicrobial peptides, for instance defensins that are continuously existing in body fluids, as well as indigenous microbiota. On the whole, the innate immunity recognizes pathogens using several pattern recognition receptors (PRRs) situated on or within the host cells. These PRRs bind to pathogen-associated molecular patterns (PAMPs) such as lipopolysaccharide (LPS), lipid-A, peptidoglycan, flagellin, and TTSS expressed by *B. pseudomallei* (41) eventually eliciting pro-inflammatory signaling pathways.

The innate immune responses to *B. pseudomallei* infection have been investigated in greater detail employing several noticeable cell types (e.g., macrophages and neutrophils) that are triggered following *B. pseudomallei* infection, together with their cell surface signaling molecules, as well as Toll-like receptors (TLRs), nucleotide-binding oligomerization domain (NOD)-like receptors (NLRs) and caspases *via* nuclear factor kappa-light-chain-enhancer of activated B cells (NF-kB) signaling pathway. Other important cytokines or interleukins include monocyte chemoattractant protein-1 (MCP-1), tumor necrosis factor alpha (TNF-α), interferon gamma (INF-γ), IL-12 and IL-8 (CXCL8).

### Evasion of Innate Immune Responses

The main forces in innate immune responses are the phagocytes, namely the macrophages and polymorphonuclear neutrophils (PMNs). With patrolling macrophages and neutrophils deployed at strategic tissue locations in the human body, *B. pseudomallei* employs versatile strategies to overcome the rivals and is equipped with multiple virulence factors in order for it to establish a successful infection.

### Overcoming Phagocytosis

The key strategy to evade potential elimination of *B. pseudomallei* by the host immune system is to circumvent their detection and phagocytosis. Different pathogens employ distinct strategies to avoid their recognition in the host. *B. pseudomallei* appears to escape from phagocytosis *via* production of a capsular polysaccharide (CPS), which abridges the deposition of C3b on their surfaces (42). CPS of *B. pseudomallei* has previously been characterized and identified as the major determinant of bacterial virulence (43). C3b is a vital component of the C3 complement system, which is pivotal to pathogenic opsonization (43). It has been demonstrated that encapsulated prototrophic *B. pseudomallei* allows poor C3b deposition rationalizing their high rates of persistence in the peripheral circulation as compared to its auxotrophic capsular-mutant counterpart. It has also been established that polymorphonuclear leukocytes (PMNLs) significantly engulfed more capsular mutants relative to capsule-producing wild types (WTs) (42). The above findings point to the role of CPS aiding in the successful evasion of the bacterium from phagocytosis.

### Subversion of NETosis and Fibrinolysis

Apart from phagocytosis, neutrophils also release neutrophilic extracellular traps (NET) (a.k.a. NETosis) upon their activation following their recruitment to the site of infection. NET, formed by neutrophilic DNA, targets bacterial virulence factors and irreversibly kills the bacteria trapped within (44). NETs are known to entrap both Gram-positive and Gram-negative bacteria. It has been demonstrated that activated neutrophils release NETS upon challenging by *B. pseudomallei* and the number of extracellular bacteria were shown to be reduced indicating the bactericidal activity of NETs. When DNase I treatment was applied to dissociate NETs, the killing of the bacteria was reduced (45). The results suggest that NETs do possess antibacterial activity against *B. pseudomallei*. The question as to whether *B. pseudomallei* is equipped with a surface endonuclease (similar to *Streptococcus pneumoniae*) (46) remain dubious as *B. pseudomallei* excretes various exoenzymes and its genome prediction was also found to contain a nuclease (47). The nuclease (if expressed) would degrade NETs to enable the bacteria to escape from killing by antimicrobial molecules present in NETs.

Fibrinolysis plays a paramount role in host immunity against invading bacteria. The fibrin clot ensures the closure of injured blood vessels preventing access to more pathogens, and concentrates and entraps intruders at a focused site preventing their systemic dissemination. During the process of fibrin clot formation, inflammatory responses appear to occur entailing phagocytic influx (48). Although it also appears that many pathogens likely interfere with the plasminogen-plasmin system for systemic dissemination and escape from phagocytes (49), there is no proof on such ability of *B. pseudomallei*. Indeed, it has been reported that urokinase-type plasminogen activator receptor (uPAR) that regulates fibrinolysis and inflammation is up-regulated upon challenging by *B. pseudomallei* in septic melioidosis and experimental melioidosis in mice (50). Even though phagocytosis of *B. pseudomallei* was impaired in uPAR knockout mice there was no difference in the fibrinolytic responses in both uPAR KO as well as WT mice. This suggests that unlike several other successful invading pathogens, *B. pseudomallei* may not interfere with the plasminogen-plasmin system for evasion from phagocytic activity.

#### Living and Thriving Within Phagocytes

One of the smartest ways of overcoming extracellular killing is to invade and adopt an intracellular lifestyle. The ability to adopt an intracellular lifestyle is one of the hallmarks of pathogenesis of *B. pseudomallei* (51). The various strategies employed by the bacterium for intracellular survival have been critically reviewed previously (52). As expected, T3SS cluster together with a few other virulence factors, namely adhesins and pilins, enhance their adhesion, invasion and intracellular survival within phagocytic and non-phagocytic cells (53).

For efficient survival and persistence, the bacteria must escape from the endocytic vesicles into the cytoplasm, which is rich in nutrients than the endocytic vesicles on gaining access into the intracellular milieu. *B. pseudomallei* will escape from endocytic vesicles within 15 minutes after internalization, according to an electron microscopy analysis (54). Research has also revealed that T3SS proteins like BopA, BsaZ, and BipD are most likely involved in the escape mechanisms (55) although the other possibilities remain unclear. Allwoods et al. (2011) (27) proposed two potential mechanisms based on the projected functional domains found in BopA: i) BopA has a carboxyl-terminal Rho GTPase inactivation domain that could purpose as a protease or acyltransferase on the vesicle. ii) BopA has a cholesterol-binding domain, which can prevent cholesterol depletion, resulting in phagolysosome or autophagolysosome formation and bacterial degradation (56).

Despite its aptitude to evade from endocytic vesicles, *B. pseudomallei* can suppress macrophage killing processes. The action of inducible nitric oxide synthase (iNOS) results in the development of the free radical species nitric oxide (NO) in macrophages necessary for the removal of intracellular pathogens (57). It was discovered that LPS of *B. pseudomallei* were responsible for lowering NO expression in macrophages as compared to LPS of *E. coli* or *Salmonella enterica* (58). This is believed to be caused by *B. pseudomallei* LPS, which prevents macrophages from secreting IFN-γ required to stimulate iNOS expression (59). Furthermore, intracellular *B. pseudomallei* can modulate the expression of a few proteins, including SOCS3 (suppressor of cytokine signaling), CIS (cytokine-inducible Src homology), and SIRP (signal regulatory protein), which together inhibit the expression of iNOS (58). These are the few efficient ways by which *B. pseudomallei’s* progress with intracellular survival within macrophages.

Intracellular bacteria can still succumb to autophagic clearance after escaping into the phagocyte cytosol, while others alter or evade autophagy to survive intracellularly (60). The survival of intracellular *B. pseudomallei* was found to be significantly reduced when infected RAW 264.7 cells treated with rapamycin (inducer of autophagy). In the control model, only a subgroup of bacteria co-localized with the autophagy marker protein, LC3, resulting in a higher number of intracellular *B. pseudomallei* that survived (56). Since the survival of BipD and BopA mutants in RAW 263.7, T3SS proteins are expected to be involved in avoiding autophagy, the cells were affected, and there was a complex degree of co-localization with LC3 and the lysosomal marker LAMP1*. B. pseudomallei* encodes a T6SS cluster-associated gene, bpss0180, which can regulate autophagy in both phagocytic and non-phagocytic host cells (61). This indicate that *B. pseudomallei* could alter autophagic attack in the host, but the underlying mechanism remain ambiguous. According to a unique theory, *B. pseudomallei* can also influence the ubiquitination pathway in host cells, where the bacteria can identify a target protein, ubiquitin. In this relation, TssM, a T6SS cluster 5 protein encoded by the bpss1512 gene was recognized in *B. pseudomallei*, which can obstruct the ubiquitination of essential signalling intermediates (62). *B. pseudomallei* also produces CHBP (Cif homolog *in B. pseudomallei*), a cyclomodulin that inhibits the eukaryotic ubiquitination pathway (27). Despite the lack of direct evidence, these findings indicate that *B. pseudomallei* may have the ability to modulate ubiquitination to avoid recognition and bacterial clearance through autophagy.

Some pathogenic bacteria may also use ubiquitination to target and suppress immune system components in the host (63). The capability of *B. pseudomallei* to degrade a transcriptional factor, ELT-2, with targeted ubiquitination targeting has been demonstrated (64). In *C. elegans*, the GATA transcriptional factor ELT-2 regulates the early immune response to an invading pathogen (65). The targeted suppression of ELT-2 appears to be restricted to infection with *B. pseudomallei*, as the protein level of ELT-2 in *C. elegans* decreased over time while remaining relatively constant when infected with other bacteria. *P. aeruginosa* is known to use T3SS and T4SS effector proteins to promote the ubiquitination of host target proteins to inactivate host defenses (66). T3SS of *B. pseudomallei* was also thought to be responsible for inducing unique host E3 ligases to target ELT-2 for ubiquitin-proteasome system (UPS) destruction (65). More research is in progress to determine the effectors and components of T3SS, as well as the mechanism involved in subverting UPS to degrade ELT-2 protein.

### Festering *B. pseudomallei* Infections

Several clinical studies have shown that chronic melioidosis can present a variety of clinical symptoms that differs from acute melioidosis. The acute disease is often linked to risk factors including diabetes, heavy alcohol use, and renal disease (9). Chronic *B. pseudomallei* infections, on the other hand, are considered less severe; however, they are more localized in specific organs or locations, and the bacteria are tough to treat in general (67, 68). Relapses are common even after antibiotic therapy, and chronic melioidosis may develop after acute symptoms have subsided, with symptoms lasting for months to years. The treatment strategies also vary for chronic infection in comparison to acute infection. Experimental evidences are documented on a higher end for acute infections rather than chronic conditions using *in vitro* melioidosis pulmonary infection models. As a result, very minor is known about the virulence mechanisms and pathology of *B. pseudomallei* chronic infections (69). However, these chronic model experiments have revealed relative resistance to *B. pseudomallei* infections, not expressing much relapses (68).

### TLR Signaling in *B. pseudomallei* Infections

TLRs belong to IL-1 receptor superfamily host transmembrane receptors which identify the conserved *B. pseudomallei* molecular trends inducing a pro-inflammatory response in concert with nuclear transcription factor (NF-B) translocation. Furthermore, TLRs have been elucidated as key PRRs in *B. pseudomallei* infection (70). Previous studies have shown the critical roles of TLR 2 and TLR4 as receptors in the host pulmonary defense (71). The host is exposed to B*. pseudomallei* lipopeptides and peptidoglycan, which activates TLR2, followed by TLR1 or TLR6. TLR4 being a significant LPS receptor, it is considered as a key molecule in defense against *B. pseudomallei* (70). However, some studies have shown that TLR-independent activation involves a functional Bsa T3SS followed by *B. pseudomallei* internalization (72). They discovered that live *B. pseudomallei* can activate TLR2 even in the absence of the co-receptor CD14 substantiating the necessity of TLR4 stimulation in the occurrence of MD2 and CD14 (73). TLR2 and TLR4 expression were activated and increased during *B. pseudomallei* infection *in vivo* and *in vitro*, according to Wiersinga and colleagues (2007) (71). Additionally, patients with septic melioidosis have higher TLR2 and TLR4 expression and activation (74) by *B. pseudomallei.* They also discovered a defensive phenotype in TLR2-deficient mice infected with *B. pseudomallei* through intranasal infection. Surprisingly, they discovered TLR2 activation but not TLR4 stimulation in transfected HEK293 cells during infection with *B. pseudomallei* LPS.

In another *in vivo* melioidosis acute mice model, Chin et al. (2010) (36) found that the expression of several TLRs (TLR2, 3, 4, 5, 6, and 7) were curbed; TLR2 was strongly expressed, while TLR4, which perceives LPS, was weakly induced. Whiteley et al., 2018 (75) found a similar expression profile *in B. pseudomallei*-infected RAW264.7 macrophages. This indicates that TLR2 is primarily responsible for the processing of signals that activate pro-inflammatory cytokines in response to *B. pseudomallei* infection and that TLR4 is not included in host defense against melioidosis *in vivo.* On the other hand, the mechanism where TLR2 signaling contributes to *B. pseudomallei* pathogenesis is still unknown. According to Chin et al. (2010) (36), up-regulation of the TLR2 facilitated signaling pathway is liable for the identification and commencement of an inflammatory response in an acute *B. pseudomallei* infection. This supports a previous finding of enhanced survival in TLR2 with reduced bacterial burden in the KO mice lung. When the main TLR adaptor signalling protein (MyD88) lacks in the host, *B. pseudomallei* infection seem to be extremely susceptible due to a decrease in the neutrophil recruitment and activation (70). TLR-mediated MyD88-dependent cell signalling appears to have a protective function, but dysregulation of TLR-mediated immune response could lead to pathogenesis and further allow to the production of septic melioidosis. TLR polymorphisms are also known to influence the host resistance to melioidosis, but the mechanisms underlying this effect remain unknown.

### NF-kB Signalling Pathway in *B. pseudomallei* Infections

At large, TNF-α, IL-1, IL-6, IL-8, and IL-12 are some of the interleukins involved in the inflammatory response, and NF-kB is a key transcription factor that modulates their expression (76). When TLRs detect *B. pseudomallei* infections, they activate the NF-kB pathway, lowering the host immune response. *B. pseudomallei* activates NF-kB in a MyD88-independent but partially NOD1 intracellular receptor-dependent manner that necessitates the activities of Cdc42 and Rac1. The role of NF-kB in infection resistance was demonstrated in a knockout mouse trial, in which mice lacking various mechanisms of the NF-kB pathway were inclined to different infections (77). Tan et al. (2010) (62) recently established that *B. pseudomallei* prevents the initiation of the NF-kB and type I IFN pathways, thus reducing host inflammatory responses. Since NF-kB is involved in the bacterial clearance *via* host’s immune response it is a fair play by many pathogenic bacteria by developing mechanisms to control the pathway to suppress host immune responses.

### NLRs and Caspases in *B. pseudomallei* Infections

Altogether, NOD1, NOD2, NLRP2, NLRP3, and class II trans-activator (CIITA) are members of the NLR gene family, which functions as an intracellular sensor of cytosolic microbial products and “hazard signals” during infection. Finally, this process stimulates caspase (CASP) cascades, leading to apoptosis with an increase in inflammatory responses, both of which are critical in regulating the intracellular *B. pseudomallei*. The NLRs, NOD1 and NOD2 were found to control the development of IL-18 after *B. pseudomallei* infection (72). IL-18 has been demonstrated to protect against *B. pseudomallei* infections in response to inflammasome signalling (71). However, *in vivo* studies demonstrated that the mice lacking inflammasome mechanisms such as ASC, caspase-1, NLRC4, and NLRP3 were reportedly more vulnerable to *B. pseudomallei* infection than the wild-type mice (78).

Different caspases were shown to be activated in response to *B. pseudomallei* infections, including the inflammatory mediator subfamily (CASP1, CASP4), the apoptotic activator (CASP2, CASP8), and the apoptotic executioner (CASP7) (36, 79). *B. pseudomallei* infections have been shown to activate CASP1, resulting in the cell death of macrophages and the development of IL-1 and IL-18 (80). Subsequent to *B. pseudomallei* infection, the CASP1 pathway influenced the IFN-production. The CASP1/mice model was found to be extremely vulnerable to *B. pseudomallei* infection with elevated bacterial loads, and their IFN-release was significantly reduced (80).

### Role of MCP-1 in *B. pseudomallei* Infections

MCP-1 is a mononuclear cell attractant and an activator that may be a key modulator of mononuclear cell migration (81). The MCP-1 gene deletion caused in a moderately impaired monocyte conscription, decreased bacterial elimination in the spleen, and increased infection susceptibility (82). This inconsistency in the expression is due to the role played by the NK cells as the primary producers of IFNc in the lungs, while epithelial cells are unable to produce IFNc. In *B. pseudomallei*-infected murine lung epithelial cells (LA-4), MCP-1 levels were found to be significantly higher than in uninfected control cells (83). In addition, Sim et al. (2009) (83) had earlier proposed that lung epithelial cells may produce pro-inflammatory cytokines and chemokines, as essential mechanisms of the innate immune response, during the early *B. pseudomallei* infection. MCP-1 upregulation in murine lung epithelial cells, on the other hand, was dependable with the earlier *in vivo* studies (84).

### Effect of TNF-α During *B. pseudomallei* Invasion

TNF- is a member of a set of cytokines that activate the acute phase reaction and is involved in systemic inflammation. These are generated by activated macrophages generate it, but can also be released by varies cell types such as CD4+ lymphocytes, NK cells, and neurons. Barnes et al. (2008) (85) demonstrated that TNF- is needed to regulate *B. pseudomallei* infection in a mouse model, in which the mice were more inclined to infection with increased number of bacterial in the spleen and liver, with elevated mortality rates in TNF-/, TNFR1, and TNFR2/mice. Increased TNF- levels in human melioidosis sepsis patients, on the other hand, have been linked to higher mortality rates (3). Furthermore, Bearss et al. (2017) (86) discovered that TNF- levels were significantly higher in the livers of BALB/c mice upon infection with *B. pseudomallei*, while levels were lesser in the liver of C57Bl/6 mice (86). This substantiates the dual role played by TNF- in *B. pseudomallei* infection associated defence and susceptibility control.

### *B. pseudomallei* Infection and Role of IFN-γ

**Figure 1:**
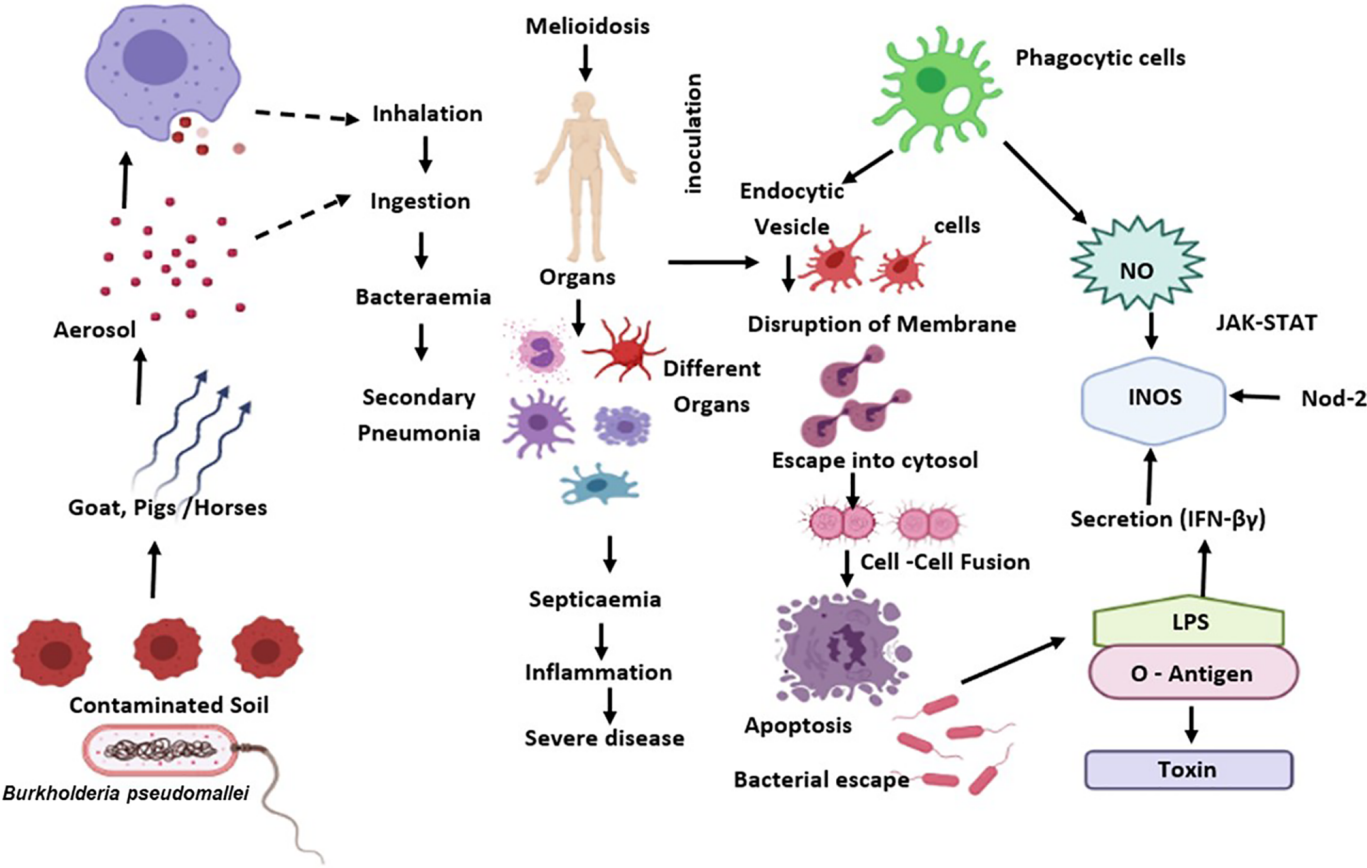
*B. pseudomallei* is diffused from the external reservoir to respiratory epithelial cells where the initial attachment initiates possibly through capsule and type IV pili. Upon invasion of epithelial cells, the T3SS-3 effectors promote in vacuolar evade and intracellular motility *via* BimA-mediated actin polymerization. Subsequent to phagocytosis, *B. pseudomallei* exits the phagosome and enters the host cytoplasm to replicates. This eventually causes the host cell death through initiation of apoptosis. The *B. pseudomallei* LPS regulates IFN secretion, which down-regulates iNOS expression and NO production. Also, *B. pseudomallei* influence the regulation of suppressor of cytokine signalling and cytokine-inducible Src homology 2-containing proteins that obstruct the JAK–STAT signalling pathway and iNOS activation.

During *B. pseudomallei* infection, IFN- is an effective regulator for generating protective immunity (87). Previous studies indicate that IFN- could regulate the proliferation of *B. pseudomallei* in macrophages, as a preliminary target after its entry *via* inhalation. Activation of IFN-γ by macrophages helps host defence by phagocyting and destroying *B. pseudomallei via* release of reactive oxygen and nitrogen intermediates (ROIs and RNIs), cytokine and chemokine, and antigen presentation to T cells (88) (**Figure 1**).

Several other immune evasion strategies exist that includes inhibition of iNOS and development of TNF-α, up-regulation of the suppressor of cytokine signalling 3 (SOCS3) and cytokine-inducible Src homology 2-containing protein (CIS) that resulted in IFN-signaling reduction (53, 89). IFN- γ is primarily generated by NK cells and stimulated CD8+ T cells (90), but these cells are not actually involved in early defence, implying that the source of IFN- is highly redundant (91). The IFN- γ response is required for resistance in mice, particularly during early infection with *B. pseudomallei*, as established by the swift death of IFN- γ KO mice and mice treated with neutralizing monoclonal antibodies (mAb) against IFN- γ (71, 91, 92). They have revealed that the IFN-γ-inducing cytokines IL-12 and IL-18, are necessary for *B. pseudomallei* infection resistance (92).

Koo and Gan (2006) (93) observed overproduction of IFN-γ in mice during unrestrained bacterial loads. Additionally, Breitbach et al. (2009) (80) also found that macrophages from mice were not as susceptible to IFN-γ stimulated killing of *B. pseudomallei*. Recently, some researchers discovered that IFN-γ development controls *B. pseudomallei* intracellular killing, which is consistently higher in IFN-γ/- mice compared to WT infected with *B. pseudomallei.* They also suggested the initiation of IFN-γ production *in vivo* has not been completely illuminated; however, higher IFN-γ production by NK cells in the lungs have been shown (94).

### Cell-to-Cell Interactions by *B. pseudomallei*

*B. pseudomallei* evades humoral and cell-mediated immunity by rapidly spreading from cell to cell. In a human melioidosis infection, *B. pseudomallei* and its main microbial virulence factors not only promotes tissue invasion and necrosis but also enable evasion of normal humoral and cell-mediated immunity by surviving within both phagocytic and non-phagocytic host cells (37, 69). *B. pseudomallei* could colonize the host cell by performing phagosomal escape into the cytoplasm, resulting in multinucleated giant cells (MNGC) (54, 61). Although the exact function of MNGC in melioidosis relapse is unknown, we believe that the bacteria are encapsulated by MNGCs, preventing exposure to circulating antibodies and B cells that surround the host cell and further failure in neutralization. MNGC can fuse with surrounding cells under suitable conditions, releasing the dormant bacterial cells with further dissemination (95).

*B. pseudomallei* also uses an immune evasion technique called actin tail formation to replicate and spreads from cell to cell (96). This process prevents bacterial cells from coming into direct contact with the immune effector cells that are present around the host cells. This process is carried out by *B. pseudomallei* using actin-based mobility, which allows the pathogen to travel very rapidly across the cytoplasm. The filaments of actin that drive the bacteria in its movement are extremely stable (97). *Rickettsia rickettsii*, which propagates by a similar mechanism as *B. pseudomallei* and can travel at 4.8 m/min in the cytoplasm, is an example of bacteria that has successfully evaded the immune system (98). Based on the other results, it can be elucidated that *B. pseudomallei* could propagate much faster inside the cytoplasm, allowing them to spread systemically and outpace the human immune system’s surveillance rate (99).

## Cell-Mediated Immunity and *B. pseudomallei*

**Figure 2:**
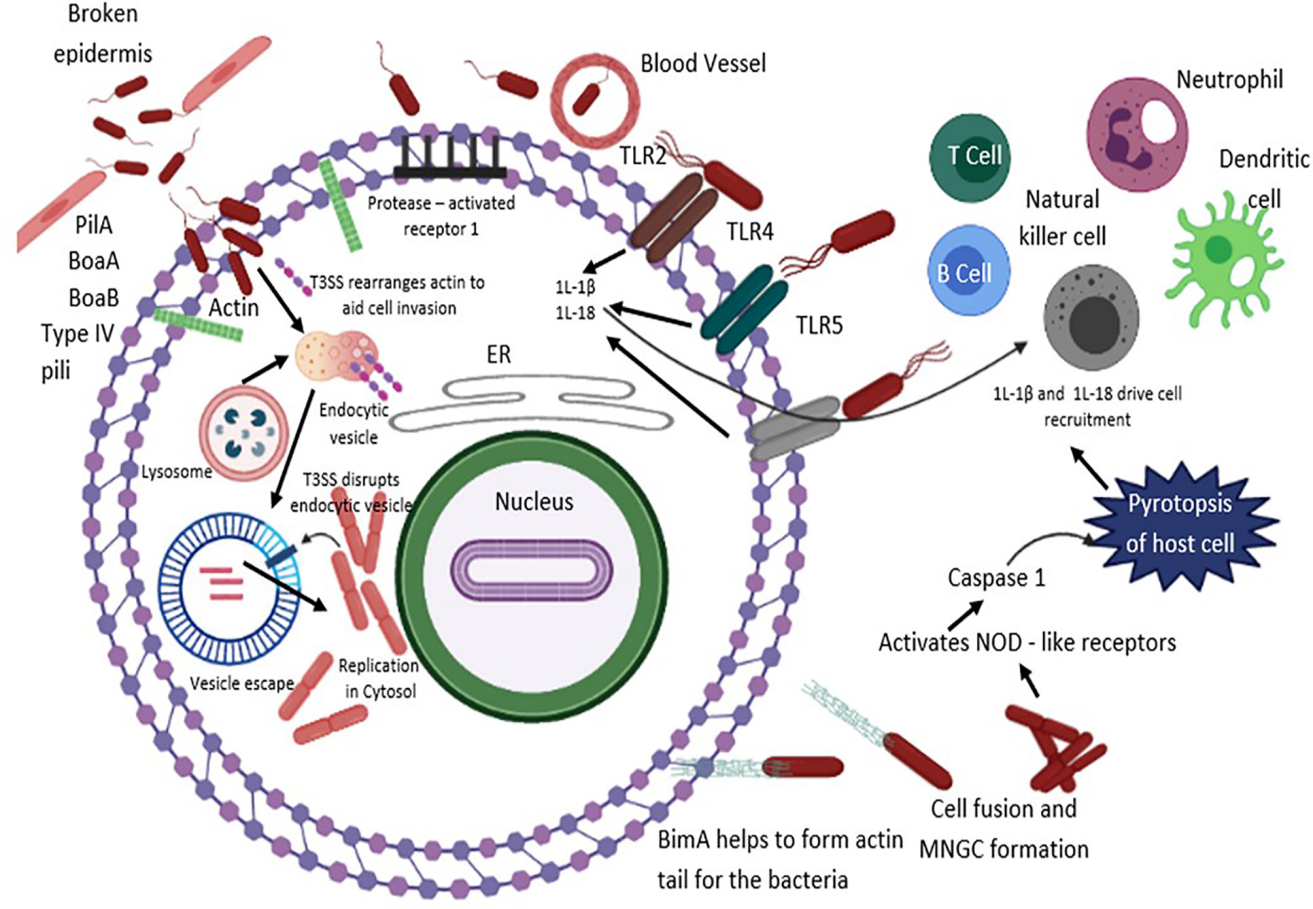
*B. pseudomallei* secretes N-acyl-homoserine lactone (AHL) signalling molecules that are implicated in coordinating attacks in contradiction of the host environment and biofilm formation. The T3SS effector proteins are vital for invasion and escape from host endosomal vesicle. Host cell entry is assisted by flagella, lipopolysaccharide (LPS), type IV pili and adhesion molecules. *B. pseudomallei* evades the vesicle and lyses the endosomal membrane using T3SS, T6SS and T2SS. Production of cationic peptides and ecotins facilitate *B. pseudomallei* to survive within an acidic endosomal environment. BopA (an effector protein) and BipD (translocator protein) obstruct sequestration in endosomal vesicles and avert microtubule-associated protein mediated autophagy. Upon entry into the cytoplasm compartment, *B. pseudomallei* replicates, and initiates the development of actin-based membrane protrusions and can pass through constant polymerization of host cell actin, thus accelerating dissemination to neighboring cells causing cell fusion and multinuclear giant cell (MNGC) formation. TLRs positioned on cell surfaces engage with the PAMPs and mediate NF-κB-induced initiation of immune responses, releasing pro-inflammatory cytokines IL-1β and IL-18 and facilitate caspase-1-mediated pyroptosis. Additionally, IL-18 warrants defensive IFNγ production, which allows recruitment of neutrophils, dendritic cells, B cells and T cells to the infection site, eventually activating the complement and coagulation cascades.

During infection, the adaptive immune response is known as the third line of defense, and it has the potential to provide specific, long-term, and highly efficient protection against vital pathogens. Adaptive immune response gets triggered upon the breach of specific external barriers and *B. pseudomallei* is likely to compromise the adaptive immune system (37). Both T- and B-cells are involved in the cellular immune response, and they work together to suppress microbial infection (100). The cytotoxic T-cell, memory B-cell, and antibodies confers the third line of protection in the human body (101) (**Figure 2**).

It is a well-known fact that cell-mediated immune responses mounted by lymphocytes activate leukocytes *via* cell-to-cell interaction or secretion of cytokines (100). The importance of CMI responses in melioidosis has become critical, as little is known about their role in the disease. Furthermore, since *B. pseudomallei* is an intracellular pathogen specific immune responses are key to antibacterial defence (16).

An avid understanding on the cellular immune response is essential for vaccine and drug delivery systems to induce the appropriate immune effectors for stimulating a defensive response and generation of specific antibodies against any pathogen (85, 102, 103). In view with this, Healey et al. (2005) (104) used dendritic cells (DC) to induce CMI responses to *B. pseudomallei* and are essential for the development of adaptive immune responses. There has also been clinical (90, 105) and experimental evidence (90, 106) to indicate that CMI may be vital for the existence of *B. pseudomallei* after infection. Individuals not affected by *B. pseudomallei* can undergo seroconversion developing a robust CMI, which may defend them from disease progression.

### T Cell-Mediated Immunity Underlying *B. pseudomallei* Infections

T-cell possess specific antigen receptors that can significantly bind to the target antigens leading to cytotoxicity. The foreign antigen must typically be recognized in combination with the self-major histocompatibility complexes (MHC) molecules to evoke T-cell immune responses (107) via expression of unique membrane molecules. The main receptors include CD4+ or CD8+ at the same time, and both help the T-cells to communicate with antigen-presenting cells (APC) by interacting with the antigen-presenting molecules. Though there are numerous reports on the role of CD4+ and CD8+ T cells in immunity to many pathogenic species (108), it still remains a ‘tip of the iceberg’ in the context of melioidosis.

Tippayawatet al. (2009) (90) found that individuals who are healthy with serological manifest of *B. pseudomallei* exposure and melioidosis patients produced CD4+ and CD8+ T cells that recognized the *B. pseudomallei* ATP-binding cassette (ABC) transporter family. A robust T-cell response might be needed for the determination of clinical melioidosis. In patients who improved from clinical melioidosis, *B. pseudomallei* reactive T-cells were stimulated and able of generating antigenic-specific gamma interferon (IFN) with the ability to thrive *in-vitro* in response to *B. pseudomallei* (3, 105).

Experimental evidences also have documented that mice infected with *B. pseudomallei* develop CMI, detected by delayed-type hypersensitivity (DTH) response with lymphocyte proliferation (88). The RAG-mice (absence of T- and B-cells) acquired infection more quickly than the C57BL/6 wild-type mice, according to a report by Haque and colleagues (2006) (109). The findings showed that primary infection with *B. pseudomallei* peaks the memory CD4+ and CD8+ T cell populations. *In vitro* studies demonstrated that the T cells isolated from *B. pseudomallei*-infected C57BL/6 mice developed Ag-specific IFN in response to re-stimulation. Further research into CD4+ or CD8+ T cell depletion revealed that CD4+ T-cell deficient mice died significantly sooner than the control mice. The CD8+ T cell depleted mice, on the other hand, had a brief survival time than the controls, but the alteration was not statistically substantial and indicated that CD4+ T cells, not CD8+ T cells, are involved in the elicited defence (86).

### T Cell Exhaustion in *B. pseudomallei* Infections

Not many studies have reported on T cell exhaustion in *B. pseudomallei* infection, which occurs due to scarce intracellular expression of IFN-γ (110). Programmed death 1 (PD-1) is a known T cell activation regulator purportedly expressed on various cell types, including T cells and myeloid cells and abundantly up-regulated during inflammation (111). Kleijn et al. (2013) (112) have stated that following exposure to a low dose of bacterial LPS, PD-1 was regulated on certain neutrophils and obstructs T cell function *via* the PD-1 pathway.

In *B. pseudomallei* infection, PD-1 mRNA was found to be elevated in the peripheral blood and also during the onset of inflammation in PMNs *in vitro*. In *in vitro* *B. pseudomallei*–infected PMNs, blocked inhibitory activity and reestablished proliferation of CD4^+^ T cells and IFN-γ proliferation were observed upon inclusion of anti–PD-1, suggesting that PD-L1 is a monitoring molecule of T cell functions and could play a major role in pathogenesis (113). Using an experimental *in vivo* mice model with persistent *B. pseudomallei* infection, See et al. (2016) (114) found a significant reduction in CD4+ T-cell functions. In addition, up-regulation of PD-1 on both CD4+ and CD8+ T cells was observed conceivably signifying that the T cells were enduring exhaustion and postulated that *B. pseudomallei* 'engineers' the exhaustion of CD4+ T cells and up-regulation of PD-1 that hypothetically enables bacterial persistence in the host.

In addition, using a mice model, See et al. (2017) (115) characterized the regulation of PD-1 and CTLA-4 levels on B cells, NK cells, and monocytes and confirmed that persistent infection with *B. pseudomallei* small colony variants (SVCs) can concomitantly cause PD-1 upregulation on B cells, NK cells and monocytes signifying host immune exhaustion. The SCVs were found to up-regulate the PD-1 on NK cells and monocytes in contrast to its WT counterpart. Based on the combined *in vivo* experiments, it was concluded that in *B. pseudomallei* SCVs infection, PD-1 was up-regulated on both adaptive and innate immune cells, whereas PD-1 up-regulation only was observed in *B. pseudomallei* WT infection (114, 115). They also speculated that SCVs initiate greater PD-1 expression to overwhelm the host immune responses and enable persistence, causing elevated bacterial burden. Recently, Menon et al. (2020) (116) demonstrated up-regulation of PD-1 on T cells *in vitro*, upon exposure to crude culture filtrate antigens of *B. pseudomallei* and suggested that this possibly contributes to deprived immune surveillance and pathogenesis.

### Humoral Immune Responses Against *B. pseudomallei*

Antibodies resulting from constant exposure to *B. pseudomallei* are normal among people living in melioidosis endemic areas. This likely could be attributed to the presence of high levels of *B. pseudomallei*-specific IgG, IgM, and IgA antibodies in melioidosis patients’ sera, which persistently raised for the length of infection (106, 117). According to a study by Khakhum et al. (2019) (116), IgG and their subclasses are inevitably significant in the detection of *B. pseudomallei* infection. Besides, a few scientific findings revealed that a discerning absence of one or more IgG sub-classes likely increases the susceptibility to recurrent infections or extend the course of an established infection. Furthermore, in melioidosis positive cases, IgG antibody exhibited a sturdier and more stable response than IgM antibody, which showed a more variable response. According to Patel et al. (2011) (88), the existence of *B. pseudomallei* in lymphoid tissues continues to induce recently maturing B cells to express IgM that can subsequently be substituted to more potent isotypes to combat the bacteria effectively. Vasu et al. (2003) (119) suggested that the levels of IgG antibodies (IgG1 or IgG2) generated against culture filtrate antigen in patients under antibiotic therapy could be indicative of the grade of infection and may be used as a standard guideline to regulate the extent of maintenance of antimicrobial therapy.

Since *B. pseudomallei* is known to be air-borne (74), the immune defence in the respiratory tract is paramount to prevention of infection. The lower respiratory tracthas a preponderance in IgG, whereas the upper respiratory fluids are enriched with secretory IgA, and with low levels of systemic IgG. Also, pathogens bound to IgA are picked up by airway macrophages via phagocytosis. As a result, we believe that *B. pseudomallei* uses macrophages as a vehicle for systemic dissemination in the host (120). Yi et al. (2019) (121) demonstrated that IgG and IgM defines the versatility of immunogenic proteins generated from the host challenged with *B. pseudomallei via* aerosol. The immune response was noticeable from the early stage of the infection and antigens elicited robust concentration for either or both of IgG and IgM.

### *B. pseudomallei* Evades Humoral Responses and Dampen Complement

Inactivation of reactive oxygen and nitrogen species, inhibition of phagolysosome development, and phagosomal escape are among the critical mechanisms employed by intracellular bacteria to evade host immune responses (122). Similarly, *B. pseudomallei* has unique characteristics that allow it to overcome its host and to meet its metabolic needs. This bacterium is currently being examined extensively from an immunological perspective to establish a void in its evasive strategies and, hopefully, find a perpetual solution to its intracellular persistence. *B. pseudomallei* causes a complement system deficiency, which dampens the humoral immunity. The capacity of *B. pseudomallei* to live in the human host is an intriguing phenomenon. *B. pseudomallei* does not lyse in human serum, according to Canadian scientists, because of its unusual CPS production, which aids in survival by inhibiting the deposition of C3b, adding to it's virulence. Further, *B. pseudomallei* decreased C3b deposition on the bacterium’s surface, rendering it resistant to phagocytosis leading to bacterial persistence in the host (25).

On the other hand, Carter and Fearon (1992) (123) showed that the B cell activation can be lowered with the co-ligation of the co-receptor and the B-cell antigen receptor (BCR), thus proving the role of complement influencing humoral responses (124). This fact was further supported by the presence of fusion proteins of multimers of C3d and lysozymes that lowered the *in-vitro* activation threshold of B cells (125). Animal models proved the relationship between the complement systems and humoral immunity control (126) substantiating the fact that if the complement-mediated immunity is decreased, the stimulus for humoral immunity will be reduced as well, leading to the elimination of *B. pseudomallei* from host tissues.

## Concluding Remarks

This review confers a better understanding on the immunological perspective behind *B. pseudomallei* infections. The organism secretes a slew of virulence proteins that amend host cell functions and further evading from the host immune response. Understanding the bacterium’s basic virulence factors and pathogenic mechanisms are essential for delving into the adaption of *B. pseudomallei* inducing a wide array of clinical manifestations. The host-parasite interactions are often influenced by both humoral and cellular immune response together with complement mediated pathways. The bacterium has also been known to evade the host’s front-line immune defenses and exploit the host’s responses to survive in the host while receiving adequate and long-term antibiotic treatment. Further elaborative elucidations on the immune mechanism of melioidosis is the need of the hour in the development and progression of novel therapeutic strategies to curb the menace of *B. pseudomallei* infections.

## Author Contributions

VM, KMV, and ES conceived the concept. VM and KMV wrote the manuscript. ES, MB, and AG co-wrote the manuscript. JV edited the manuscript. All authors contributed to the article and approved the submitted version.

## Funding

We thank University of Malaya Impact-Oriented Interdisciplinary Research Grant (IIRG019-2019) by Ministry of Higher Education - University of Malaya and Frontiers Research Grant (FG012-17AFR) for the funding. ES is supported by the Department of Science and Technology-Science and Engineering Research Board, Government of India (Grant Number CRG/2019/006096). The funders had no role in the study design, data collection and analysis or the decision to publish or preparation of the manuscript.

## Conflict of Interest

The authors declare that the research was conducted in the absence of any commercial or financial relationships that could be construed as a potential conflict of interest.

## Publisher’s Note

All claims expressed in this article are solely those of the authors and do not necessarily represent those of their affiliated organizations, or those of the publisher, the editors and the reviewers. Any product that may be evaluated in this article, or claim that may be made by its manufacturer, is not guaranteed or endorsed by the publisher.
